# Face Detection in Nighttime Images Using Visible-Light Camera Sensors with Two-Step Faster Region-Based Convolutional Neural Network

**DOI:** 10.3390/s18092995

**Published:** 2018-09-07

**Authors:** Se Woon Cho, Na Rae Baek, Min Cheol Kim, Ja Hyung Koo, Jong Hyun Kim, Kang Ryoung Park

**Affiliations:** Division of Electronics and Electrical Engineering, Dongguk University, 30 Pil-dong-ro 1-gil, Jung-gu, Seoul 04620, Korea; jsu319@naver.com (S.W.C.); naris27@dongguk.edu (N.R.B.); mincheol9166@naver.com (M.C.K.); koo6190@naver.com (J.H.K.); zzingae@dongguk.edu (J.H.K.)

**Keywords:** surveillance camera, visible-light camera, deep learning, nighttime face detection

## Abstract

Conventional nighttime face detection studies mostly use near-infrared (NIR) light cameras or thermal cameras, which are robust to environmental illumination variation and low illumination. However, for the NIR camera, it is difficult to adjust the intensity and angle of the additional NIR illuminator according to its distance from an object. As for the thermal camera, it is expensive to use as a surveillance camera. For these reasons, we propose a nighttime face detection method based on deep learning using a single visible-light camera. In a long-distance night image, it is difficult to detect faces directly from the entire image due to noise and image blur. Therefore, we propose Two-Step Faster region-based convolutional neural network (R-CNN) based on the image preprocessed by histogram equalization (HE). As a two-step scheme, our method sequentially performs the detectors of body and face areas, and locates the face inside a limited body area. By using our two-step method, the processing time by Faster R-CNN can be reduced while maintaining the accuracy of face detection by Faster R-CNN. Using a self-constructed database called Dongguk Nighttime Face Detection database (DNFD-DB1) and an open database of Fudan University, we proved that the proposed method performs better compared to other existing face detectors. In addition, the proposed Two-Step Faster R-CNN outperformed single Faster R-CNN and our method with HE showed higher accuracies than those without our preprocessing in nighttime face detection.

## 1. Introduction

Existing studies of face detection are conducted mainly on visible-light images that are captured during daytime. The adaptive boosting (adaboost) [1] algorithm, which is the first developed algorithm in the field of face detection, can perform face detection in real time. This is followed by face detection methods based on hand-crafted features, such as histogram of oriented gradients (HOG) and local binary pattern (LBP) [2,3,4,5]. In recent years, as the performance of graphic processing units (GPU) has improved, the convolutional neural network (CNN) has received attention, and therefore, various CNN-based face detection methods have been actively researched. However, as most face detection methods use a database with images captured using a visible-light camera, it is difficult to detect faces at nighttime when the intensity of illumination is low. In intelligent surveillance systems, nighttime face detection research is an important challenge because it can be used to prevent crimes that occur at night or to arrest suspects who have committed crimes at night. There are two crime scenarios where nighttime face detection can be used. The first one is pre-crime prevention. In the current system, the face images of criminal are analyzed after a crime occurs based on manual or semi-automatic segmentation of face image. This is post-crime prevention. However, our system can be used for pre-crime prevention. For example, if the face of a criminal or terrorist on watch-list at nighttime can be automatically detected and recognized in real-time, it is easier to arrest them and prevent crime in advance (pre-crime prevention). The second one is to reduce the time for checking the face of a criminal or terrorist on watch-list at nighttime in huge numbers of images captured by camera. Even in the case of post-crime prevention, human administrator or police should check the face in the huge numbers of images captured by camera, and manual or semi-automatic segmentation of face image takes much time and gives large burden to human administrator or police. By using our automatic algorithm to detect human face at nighttime, the time to check the huge numbers of images can be tremendously reduced.

To solve this problem, a near-infrared (NIR) light camera or a thermal camera has been used in most existing nighttime face detection studies [6,7,8,9,10,11,12,13]. In contrast to the visible-light camera, NIR and thermal cameras use infrared (IR) wavelengths; therefore, they are robust to ambient lighting changes and low intensity of illumination. However, in the case of the NIR camera, it is difficult to adjust the intensity and angle of the NIR illuminator according to its distance from the object. Furthermore, it is difficult to use the NIR illuminator if the object is extremely far or the object is in a relatively large area. In the case of the thermal camera, the price is high; hence, it is not practical to use it as a general surveillance camera in various environments.

Considering the above factors, in this study, we propose the first approach for nighttime face detection method based on CNN by using a single visible-light camera. The face image in high intensity condition can be acquired with artificial light even at nighttime. Therefore, we deal with only the case that the face image in low intensity condition is obtained without artificial light at nighttime in this research. Because a possible number of facial features are reduced in the images acquired at nighttime, we use a histogram equalization (HE) as a preprocessing. The contrast between face and background is increased through HE processing. Then, we perform a two-step detection process using Two-Step Faster region-based CNN (R-CNN). We adopt the Faster R-CNN proposed in [14] as the form of sequential operation in our research. In detail, the first step is to detect the body in the input image using the Faster R-CNN, and the second step is to detect the face in the detected upper body area. Based on previous research in which Faster R-CNN shows higher detection accuracy than other deep learning-based detectors [15], we adopted the Faster R-CNN in our research. However, because the processing time by the Faster R-CNN is larger than those by other detectors [15], we propose the two-step detection method to enhancing the processing speed of face detection. In our experiments, we demonstrate that the face detection performance is improved by limiting the face detection area to the upper body through the two-step detection process. In addition, we confirm that the proposed method shows a better performance than existing face detection methods [1,16,17,18,19] by comparing their results using Dongguk Nighttime Face Detection database (DNFD-DB1) and the open database of Fudan University.

This paper is organized as follows. In Section 2, we present an analysis of existing nighttime face detection studies. The contribution of this study is described in Section 3, and the details of the proposed method are explained in Section 4. The experimental results and analysis are presented in Section 5, and, finally, Section 6 concludes the study.

## 2. Related Works

There are two main methods in conventional nighttime face detection studies: multiple camera-based method and single camera-based method. The dual-band system in [6], which is a multiple camera-based method, detects faces using two cameras, namely an NIR camera and a short-wave IR (SWIR) camera. The difference in images is obtained through fusion (weighted difference) between the two camera images and detected face by applying a final threshold. However, with this method, it is difficult to adjust the intensity and angle of the IR illuminator according to the environment and calibration between the two cameras is required.

Because of this problem, the single camera-based method, which does not require calibration between cameras, has been studied. Among the existing single camera-based methods, the authors in [7,8,9] conducted face detection using only one thermal camera. Zin et al. proposed three face detection methods, among which the performance of the nighttime face detection using a multi-slit method was the highest [7]. However, if the position and angle of the camera change, the parameters need to be updated. The authors in [8,9] used adaboost algorithms based on various hand-crafted features. Agrawal et al. [8] developed a face detection that performs a decision-level fusion of two different adaboost results by using Haar-like features and LBP features, respectively. Both adaboosts are trained as thermal images, and the performance of the decision-level fusion that outputs only a small detection box out of two detection boxes of the two adaboosts is the highest. Ma et al. used new features considering the characteristics of the face region in the thermal image [9]. This method creates a feature pool by combining the absolute multiblock local ternary pattern (AMB-LTP), which is created by extending existing multiblock LBP features [3], with Haar-like and histogram of oriented gradients (HOG) features. Through the adaboost training algorithm, a cascade classifier is constructed by selecting optimal features among the features that were mixed while the system is learning thermal image patches (faces and non-faces). Through a comparative experiment in [9], it was proved that nighttime face detection using mixed features of Haar-like, HOG, and AMB-LTP is superior to the conventional face detection using HOG features and Haar-like features. 

The researchers in [7,8,9] used a thermal camera that is robust to illumination changes to detect faces at night. However, since a thermal camera is expensive, it is not practical to use it as a surveillance camera in various environments. In addition, face detection is not as effective in an environment where the background temperature is similar to human temperature.

Because of these drawbacks, studies using NIR or SWIR cameras have been conducted [10,11,12,13]. NIR or SWIR cameras are robust to illumination changes and cost less than thermal cameras. In [10,11], the face of a person inside a vehicle is detected during day and night. Murphy-Chutorian et al. [10] detected a driver’s face using an NIR camera installed in the vehicle at night. Hao et al. [11] detected the face of a person inside a vehicle by using an NIR camera installed outside the vehicle. The face detection process is performed in two steps. First, the windshield region, in which the face is located, is segmented using the optimal elongated directional operators and Hough transform method, and extracted as the region of interest (ROI). Second, the face is then detected within the ROI using the adaboost algorithm based on extended features [20]. Lemoff et al. [12] conducted tracking and recognition of people at a distance during day and night using their own tactical imager for night/day extended range surveillance (TINDERS) system. Hu et al. [13] used a model that combines an adaboost and the fully convolutional network (FCN) [21] to detect the face of a person sleeping at night. In [22], they analyzed the performance of the Viola Jones face detector with NIR images. Their experimental results show that the detection accuracy diminished according to the increase of standoff distance. The authors in [10,11,12,13] solved the problem of face detection at low intensity of illumination by using an NIR or SWIR camera. However, it is difficult to adaptively adjust the intensity and angle of the IR illuminator of such cameras.

Owing to the problems of using thermal, NIR, and SWIR cameras, face detection studies using visible-light cameras, which are inexpensive and require no additional equipment, have been actively conducted [23,24,25,26]. The authors in [23,24] studied face detection using a skin segmentation method in various illumination environments. Ojo et al. [23] used a hybrid skin segmentation method by the rule-based technique in [27]. Chow et al. [24] proposed a face detection method using a region-based skin color segmentation. However, the methods [23,24] have difficulty detecting faces at night when there is low color information and the noise level is high. Li et al. [25] conducted nighttime real-time face detection research using the promotion normalized pixel difference (PRO-NPD) feature [16], which presents the ratio of the difference to the sum between two pixel values. Moazzam et al. [26] detected faces using the genetic algorithm (GA) [28] in complex lighting conditions. This method, however, has a disadvantage in that the preprocessing is complicated and only one face is found among multiple faces in the image. The authors of [29,30] proposed image enhancement methods to improve face detection performance. Laytner et al. [29] proposed a method for improving the performance of the adaboost algorithm based on Haar-like features. In contrast to conventional HE, the brightness of a pixel is nonlinearly transformed and does not depend on the rest of the image; thus, the brightness of the face is always converted correctly. Rizwan et al. [30] proposed a local enhancement method to improve the face detection performance in images with low intensity of illumination and low contrast. In previous research [31], they demonstrated the effectiveness of their proposed method, named run-time adaptive sliding window (RASW), to enhance the performance of the Viola-Jones detector. Most existing face detection studies using visible-light cameras are performed in environments with a low intensity of illumination in which external light is available to some extent or in which the object is close; therefore, these methods cannot be applied to environments where almost no external light is present or the object is very far.

In previous studies [32,33], researchers proposed the method of nighttime face recognition at large standoff based on the heterogeneous face matching between NIR and visible light face images. In [34], they presented cross-spectral face recognition method between the face images by SWIR and visible light cameras based on the bank of Gabor filters, simplified Weber local descriptor, and LBP. However, the face regions in this research are manually detected, and the focus is face recognition instead of face detection [32,33,34]. Although the authors proposed the system for heterogeneous matching between NIR and visible light face images, the face regions were detected by commercial software whose algorithm was not unveiled and manually [35]. Therefore, their method also focused on face recognition instead of face detection.

Table 1 presents a comparison between the proposed method and previous methods for nighttime face detection.

## 3. Contributions

In this study, we propose the first face detection method using CNN for visible-light images taken at night to address the problems encountered in existing studies. Our study has four main contributions that differ from previous studies:

- This is the first face detection study using CNN for visible-light images taken at nighttime. Through CNN, our method automatically extracts features from nighttime images with high noise and blur levels, and it shows a high detection performance. Moreover, to improve the nighttime face detection performance, we use the HE method as preprocessing to increase both the contrast of images and the visibility of faces.
- The Faster R-CNN model uses anchor boxes of various scales and aspect ratios to detect various types of objects; in this study, however, we use only anchor boxes of appropriate scales and aspect ratios to enhance the learning optimization speed and accuracy to detect faces at nighttime and in remote environments.
- Because it is difficult to find the face immediately at night with low intensity of illumination and contrast, our method uses the step-1 Faster R-CNN to first detect the body area, because it has a larger size compared to the face, to increase the detection rate. Our method improves the detection accuracy by locating the face with the Two-Step Faster R-CNN by setting the upper body region of the found body as ROI.
- We form DNFD-DB1 and Two-Step Faster R-CNN models from the images acquired with a single visible-light camera at night, and make them available for use by other researchers [36].

## 4. Proposed Method

### 4.1. Overview of the Proposed Approach

**Figure 1:**
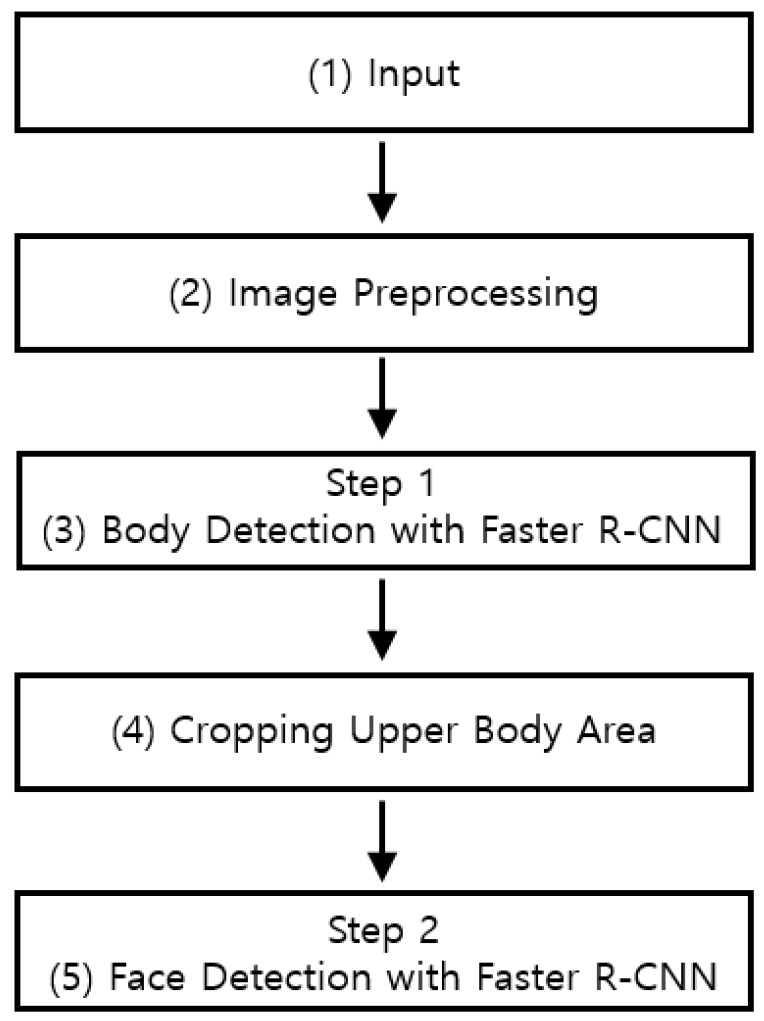
Flowchart of the proposed method.

Figure 1 is a schematic of the proposed method. The input is an image of many people captured with a visible-light camera at night. As the intensity of illumination at nighttime is low, the contrast between the face and the background is also low. CNN, which automatically extracts features from the input image, has a low contrast; thus, a high performance for blurred images is not expected. Therefore, the HE method is used in Step (2) as a preprocessing step. When HE is applied, noise is increased, but the brightness value is normalized, and therefore, the contrast of the image is also increased [37,38,39,40]. The image through HE in the preprocessing step becomes the input of Step (3), which is the body detection step. In the body detection step, the body region is detected using a Faster R-CNN model that utilizes visual geometry group (VGG) Net-16 [41], which is initially pretrained with ImageNet dataset [42]. The reason for detecting the body first without directly detecting the face is that the human body is easier to detect owing to its larger area compared to the face. Furthermore, because the face is located in the upper body region, the detection region can be limited to reduce detection errors. After the body region is detected, the upper body region where the face is likely to be located is then cropped in Step (4), which is then used as the input of Step (5). In Step (5), the final facial region is detected using the Faster R-CNN model that utilizes the pretrained VGG face-16 [43].

### 4.2. Image Preprocessing

**Figure 2:**
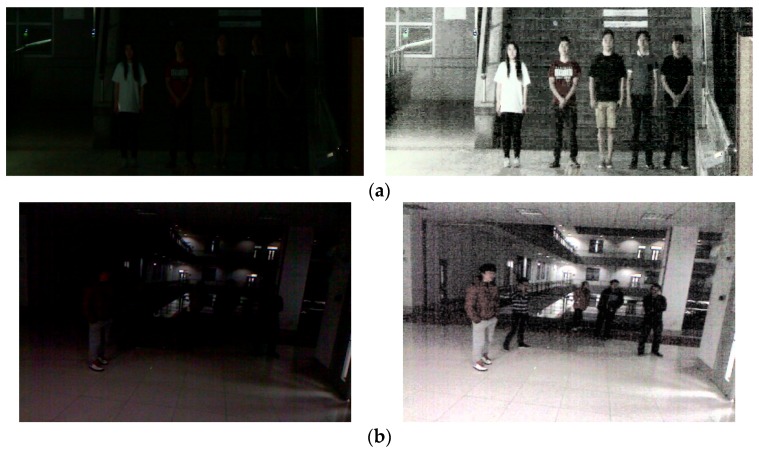
Example images for histogram equalization processing from: (**a**) Dongguk Nighttime Face Detection database (DNFD-DB1); and (**b**) open database of Fudan University. In (**a**,**b**), the left and right images show the original and histogram equalization (HE)-processed images, respectively.

Detecting faces in images taken with a visible-light camera at night is a difficult problem. Figure 2a,b shows that night vision images taken with a visible-light camera have a low intensity of illumination and lack of color information resulting in low contrast and low visibility. In this study, the HE method is used as a preprocessing process to solve this problem. The HE method obtains the result image where brightness and contrast are increased compared to those of original image. For that, the histograms converged in the range of low gray level are nonlinearly spread out in the wider range of gray level based on the normalized summation of histogram [44]. Usually, the HE method shows better performance to enhance the brightness and contrast of whole image, whereas image stretching shows better performance to improve those of local area in image [44]. 

Conventional HE methods convert the RGB image into a gray image. However, there is little color information in the night image, and color information should be used to improve the detection performance of the Two-Step Faster R-CNN. To maintain color information when HE is applied, YCbCr color space is used [44]. In the YCbCr color space, Y is a luminance component, and Cb and Cr are chrominance components. After the original night RGB image is converted to YCbCr, HE is applied to Y channel only among three channels, and the remaining channel values remain unchanged. Finally, the YCbCr image is converted into an RGB image and is then used as an input to the Two-Step Faster R-CNN. In the right images of Figure 2a,b, the contrast of the face and the background is higher than that of the left images, and the visibility of the face is also higher.

### 4.3. Two-Step Faster Region-Based Convolutional Neural Network (R-CNN) 

#### 4.3.1. Details of Faster Region-Based Convolutional Neural Network

**Figure 3:**
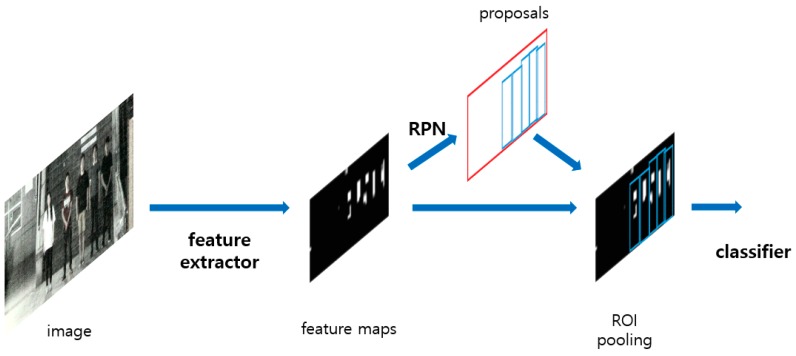
Process flow of Faster R-CNN network. RPN: region proposal network; ROI: region of interest.

The Two-Step Faster R-CNN model applied in this study uses two types of Faster R-CNNs: one to detect the body and the other to detect the face. The details of the Faster R-CNN [14] used in each stage are described thoroughly in Section 4.3.2 and Section 4.3.3. In this section, the structure of the Faster R-CNN network is described. As shown in Figure 3, when the HE-processed image is used as input, feature maps are created through the feature extractor. These feature maps are shared by the region proposal network (RPN) and the classifier. Region proposal network generates region proposals that are to be detected in the input feature maps. The classifier receives the shared feature maps and the region proposals, which are the outputs of the RPN, and generates the probability and coordinates of the finally detected faces. Thus, the Faster R-CNN can be roughly divided into three networks, namely the feature extractor, RPN, and classifier. The structure of each network is analyzed in detail.

The feature extractor in Table A1 of Appendix A is a network that automatically extracts features in an image and generates feature maps through convolutional filters. As the first step of the body and face detection in the Faster R-CNN network, the feature extractor is the most important network because it is essential for a classifier to extract good features to classify the detection boxes well. The feature extractor used in this study consists of the parts before the last max pooling layer of VGG Net-16 [41]. In Table A1 of Appendix A, the feature extractor consists of 13 convolutional layers and Relu layers, and 4 max-pooling layers. In Table A1 of Appendix A, the input image is an RGB image having a size of 300 × 800 × 3. The image passes through 13 convolutional layers and 4 max pooling layers, and finally 19 × 50 × 512 feature maps are generated. The output feature maps are then shared with RPN and classifier inputs. The RPN in Table A2 of Appendix A is the network that generates region proposals of an object with the feature maps, which are the outputs of a feature extractor, as inputs. In Table A2 of Appendix A, the RPN is an FCN consisting of a 3 × 3 convolutional layer (Conv6) and two sibling 1 × 1 convolutional layers (classification layer and regression layer). There are nine different anchor boxes in the center of the 3 × 3 sliding window [14]. In the classification layer, the object and the background score of all anchor boxes on the feature maps are generated and, in the regression layer, a bounding box regression vector [45] is generated. The bounding box regression vectors in Equations (1) and (2) are parameters that transform anchor boxes into proposal boxes.

(1) $$t_{x}=\frac{x_{proposal}-x_{anchor}}{w_{anchor}},\;t_{y}=\frac{y_{proposal}-y_{anchor}}{h_{anchor}}.$$

(2) $$t_{w}=\log\left(\frac{w_{proposal}}{w_{anchor}}\right),\;t_{h}=\log\left(\frac{h_{proposal}}{h_{anchor}}\right).$$

In Equations (1) and (2), *x*, *y*, *w*, and *h* represent the center coordinates of the box, width, and height, respectively. $x_{proposal}$ and $x_{anchor}$ are the center coordinates *x* of the proposal box and anchor box, respectively, and the same rule applies to *y*, *w*, and *h*. t_x_, t_y_, t_w_, and t_h_ are bounding box regression vector values, and RPN is trained to obtain an output of the regression layer. Anchor boxes ($x_{anchor}$, $y_{anchor}$, $w_{anchor}$, and $h_{anchor}$) are transformed into proposal boxes ($x_{proposal}$, $y_{proposal}$, $w_{proposal}$, and $h_{proposal}$) through t_x_, t_y_, t_w_, and t_h_ values. Not all generated proposal boxes are used. Only the top 300 boxes among the remaining boxes are selected to be the region proposal boxes, which then become the inputs for the classifier. The classifier in Table A3 of Appendix A takes the shared feature maps and the region proposals that are the outputs of RPN as inputs to generate the two-class (object and background) probability and bounding box regression vector of the proposal box. In Table A3 of Appendix A, the classifier consists of a ROI pooling layer, two fully connected layers (Fc6 and Fc7) and two sibling fully connected layers (classification layer and regression layer).

#### 4.3.2. Step 1 Body Detection with Faster Region-based Convolutional Neural Network

**Figure 4:**
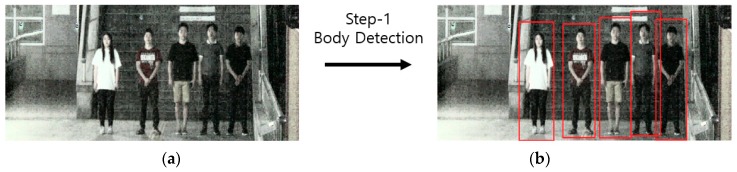
Body detection of step-1 Faster R-CNN: (**a**) input image; and (**b**) image with body detection results.

**Figure 5:**
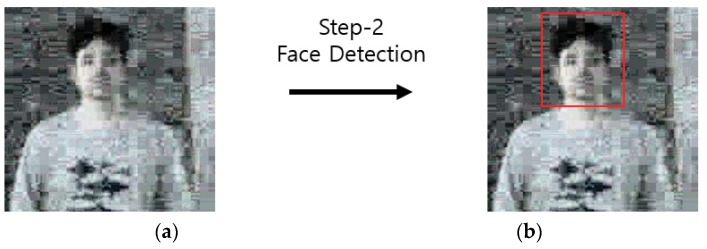
Face detection of step-2 Faster R-CNN: (**a**) upper body region; and (**b**) face detection result.

As a preprocessing process, HE is performed on the night image to enhance the visibility by increasing the contrast between the face and the background, but the face detection is still not easy because the noise level is also increased. To solve this problem, a body is first detected without directly detecting a face in an input image as shown in Figure 4. As described above, because the body region is larger than the face region, the detection error can be reduced and the accuracy can be improved by setting the upper body region, in which the face is normally located, as ROI as shown in Figure 5.

**Figure 6:**
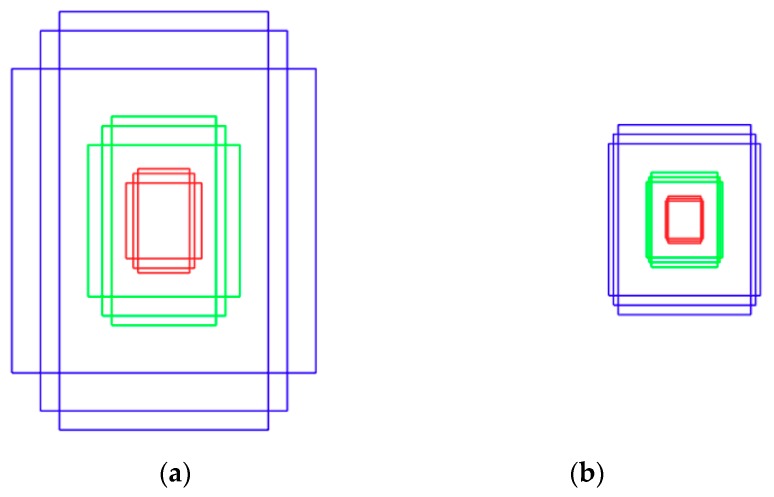
Nine different anchor boxes in Two-Step Faster R-CNN: (**a**) anchor boxes used in step-1 Faster R-CNN; and (**b**) anchor boxes used in step-2 Faster R-CNN. (Boxes with the same color in each image have the same scale).

To detect the body at night, a step-1 Faster R-CNN, where the existing Faster R-CNN [14] is modified into two classes (body and background), is used. Because the preprocessed night image contains high levels of noise and blur, the part until the last max pooling layer of the ImageNet pretrained VGG Net-16 [41] is used in the feature extractor of the step-1 Faster R-CNN. Among the existing state-of-the-art CNN models, the model that fine-tuned VGG Net-16 [41] was found to be robust and exhibited high performance in various factors including noise, blur, and illumination variations [46,47]. Therefore, VGG Net-16 is used in this study. Figure 6a shows the anchor boxes used to generate region proposals of the body in RPN of the step-1 Faster R-CNN. Because it is essential to produce good region proposals for high detection performance, it is important to use appropriate scales and anchor boxes of appropriate aspect ratios. The existing Faster R-CNN [14] uses nine anchor boxes of three scales and three aspect ratios to detect various objects. In Step 1 of this study, longitudinal-shaped boxes are used because the body of a standing person is to be detected; nine different anchor boxes of three scales (128 × 128, 256 × 256, and 512 × 512) and three aspect ratios (1:1, 1:1.5, and 1:2) are used to improve the learning optimization speed and accuracy.

#### 4.3.3. Step 2 Face Detection with Faster Region-based Convolutional Neural Network

In the body detection step, the body is detected in the entire input image, but in the face detection step, images obtained by cropping the upper body region in the previously detected body region are used as inputs. The size of the upper body image varies depending on the detected body region, and the width of the upper body image is rescaled to 240 pixels as it would be difficult to detect when the size of the face is extremely small. To detect faces in the upper body image, in the step-2 Faster R-CNN, just as in the step-1 Faster R-CNN, the existing Faster R-CNN is also modified to two classes (face and background) [14]. In the case of step-2 Faster R-CNN, the part before the last max pooling layer of the pretrained VGG face-16 [43] is used as a feature extractor, whereas original Faster R-CNN uses the pretrained VGG Net-16 [41] for the feature extractor [14]. By using the VGG face-16 model [43], which is robust against noise and blur and pretrained with large-scale face datasets, the speed and accuracy of learning optimization for fine-tuning nighttime facial images are enhanced.

Figure 6b shows the anchor boxes used to generate region proposals of the face in RPN of the step-2 Faster R-CNN. The existing Faster R-CNN [14] uses nine anchor boxes of three scales and three aspect ratios to detect various objects. In Step 2, nine different anchor boxes of three scales (64 × 64, 128 × 128, and 256 × 256) and three aspect ratios (1:1, 1:1.2, and 1:4) are used, considering the size and ratio of the face.

#### 4.3.4. Differences between Original Faster R-CNN and Our Two-Step Faster R-CNN

In this subsection, we summarize the five differences between original Faster R-CNN [14] and our Two-Step Faster R-CNN as follows.

- The existing Faster R-CNN [14] uses nine anchor boxes of three scales and three aspect ratios to detect various objects. In our Step 1 body detection with Faster R-CNN (Section 4.3.2), longitudinal shape of boxes are used because the body of a standing person is to be detected; nine different anchor boxes of three scales (128 × 128, 256 × 256, and 512 × 512) and three aspect ratios (1:1, 1:1.5, and 1:2) are used as shown in Figure 6a to improve the learning optimization speed and accuracy.
- In our two-step face detection with Faster R-CNN (Section 4.3.3), to detect faces in the upper body image, just as in the step-1 Faster R-CNN, the existing Faster R-CNN is also modified to two classes (face and background), whereas original Faster R-CNN is used for the classification of 21 classes [14].
- In our two-step face detection with Faster R-CNN (Section 4.3.3), the part before the last max pooling layer of the pretrained VGG face-16 [43] is used as a feature extractor, whereas original Faster R-CNN uses the pretrained VGG Net-16 [41] for the feature extractor [14].
- The existing Faster R-CNN [14] uses nine anchor boxes of three scales and three aspect ratios to detect various objects. In our two-step face detection with Faster R-CNN (Section 4.3.3), nine different anchor boxes of three scales (64 × 64, 128 × 128, and 256 × 256) and three aspect ratios (1:1, 1:1.2, and 1:4) are used as shown in Figure 6b, considering the size and ratio of the face.
- As a two-step scheme, our method sequentially performs the detections of body and face areas, and locates the face inside a limited body area. By using our two-step-based method, the processing time by original Faster R-CNN can be reduced while maintaining the accuracy of face detection by Faster R-CNN.

## 5. Experimental Results and Analysis

### 5.1. Experimental Database and Environment

The performance of the Two-Step Faster R-CNN was measured using DNFD-DB1 [36], which was constructed as the first database in this study. DNFD-DB1 is a self-constructed database acquired through a fixed single visible-light camera [48] at a distance of approximately 20–22 m at night. The resolution of the camera is 1600 × 1200 pixels, but the image is cropped to the average adult height, which is approximately 600. A total of 2002 images of 20 different people were prepared, and there are 4–6 people in each frame. Data augmentation was performed by applying a horizontal flip only to increase the number of images in the training of Two-Step Faster R-CNN.

In our experiments, we used both self-collected database of DNFD-DB1 (shown in Table 2) and the open database of Fudan University [49]. Training and testing were performed based on two-fold cross validation. For example, with DNFD-DB1, the data of 20 people were divided into two subsets of 10 people, as shown in Table 2. In the case of the first fold, the augmented images (1696 images of Table 2) of Subset 1 were used for training, whereas the original images (1154 images of Table 2) of Subset 2 were used for testing. In the case of the second fold, the augmented images (2308 images of Table 2) of Subset 2 were used for training whereas the original images (848 images of Table 2) of Subset 1 were used for testing. From these procedures, two testing accuracies were obtained and average value of these two accuracies was used as final accuracy.

To show the robustness of our method to these kinds of databases, our Two-Step Faster R-CNN trained with DNFD-DB1 was fine-tuned using training set images in the open database of Fudan University, and the accuracies were measured with the testing set images in the open database. For the training with training dataset and measuring accuracies with testing dataset, the regions of faces and bodies were manually extracted and presented both in the training and testing datasets.

**Figure 7:**
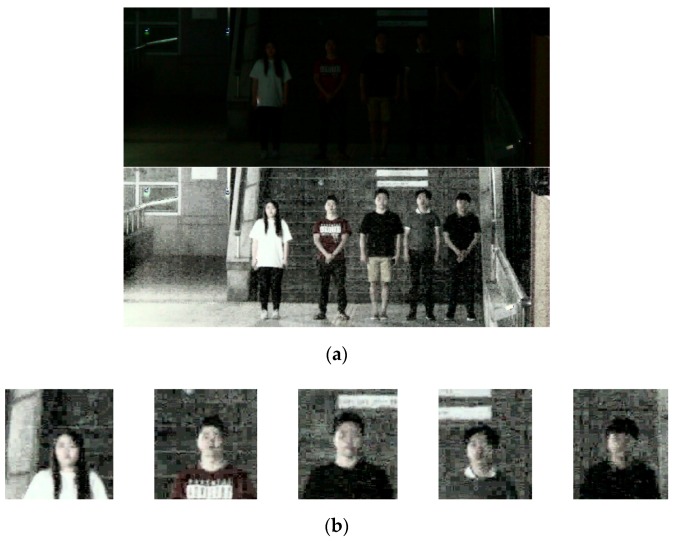
Examples of images in DNFD-DB1 used for experiments: (**a**) images of DNFD-DB1 (the original image is on the left and the HE-processed image is on the right); and (**b**) upper body images of DNFD-DB1.

Figure 7a shows the original image and HE-processed image of DNFD-DB1, and Figure 7b shows images obtained by cropping only the upper body region of the body detected by step-1 Faster R-CNN. The right image in Figure 7a is used for the learning of step-1 Faster R-CNN, and the images in Figure 7b are used for the learning of Two-Step Faster R-CNN.

Table 2 summarizes the numbers of original images and augmented images, the number of face annotations, resolution, face size (width and height), and the database environment of the two subsets of DNFD-DB1 used in the experiment.

Each experiment used a desktop computer (Intel Core i7-7700 4-core CPU at 3.6 GHz with 16 GB main memory) equipped with an NVIDIA GeForce GTX 1070 GPU (1920 cores and a graphical memory of 8 GB) [50]. We implemented each algorithm for training and testing by Matlab Caffe (version 1) [51] with Matlab (version 2017a) (MathWorks, Natick, MA, USA) [52], and compute unified device architecture (CUDA) (version 8.0) [53] with CUDA deep neural network library (CUDNN) (version 5.1) [54].

### 5.2. Training of Two-Step Faster R-CNN

**Figure 8:**
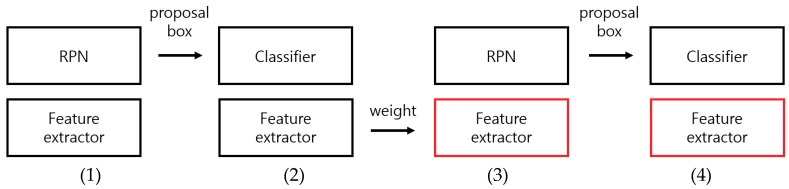
Schematic of the four-step alternating training. (1)–(4) are the steps for learning Faster R-CNN. The feature extractors in Steps (1) and (2) are initialized to weights of VGG Net-16, which are pretrained with ImageNet dataset by using the end-to-end learning. The feature extractors in Steps (3) and (4) use the weights of the feature extractor learned in Step (2), and only the RPN and classifier are fine-tuned (the red box indicates a network that does not learn).

The learning method of the Two-Step Faster R-CNN used in this study is a four-step alternating training, which is the learning method of the existing Faster R-CNN [14]. As shown in Figure 8, the feature extractor and RPN are learned by using the end-to-end process in Step (1). In Step (2), the feature extractor and the classifier are learned by using the end-to-end process and the proposal boxes generated from the learned RPN. The feature extractors in Steps (1) and (2) are not shared, and each feature extractor is initialized to the weight of VGG Net-16 [41], which is pretrained with the ImageNet dataset. In the case of Two-Step Faster R-CNN, the feature extractor is initialized using the pretrained VGG face-16 [43] in Steps (1) and (2). From Step (3), the feature extractor is shared and the weights of the feature extractor learned in Step (2) are used as they are, and only the RPN is fine-tuned. Finally, in Step (4), the shared feature extractor is fixed, and only the classifier is fine-tuned to finish the learning. The procedure shown in Figure 8 is performed separately for each model of the Two-Step Faster R-CNN. The inputs for step-1 Faster R-CNN include the entire HE-processed images, and the inputs for step-two are the images obtained by cropping only the upper body region in the body detected in the previous step.

The stochastic gradient descent method (SGD) [55] is used to train the Two-Step Faster R-CNN, and the hyperparameters, i.e., momentum, weight decay, and learning late, are set as 0.9, 0.0005, and 0.001, respectively. A total of 40,000 SGD iterations are performed only in the training of the step-1 classifier, and 80,000 SGD iterations are performed for the rest.

Equation (3) is a multitask loss function [56], and the RPN and classifier of Two-Step Faster R-CNN are trained to minimize this loss function.

(3) $$L\left(p_{i},p_{i}{}^{*},t_{i},t_{i}{}^{*}\right)=\frac{\sum_{i}L_{\mathrm{cls}}\left(p_{i},p_{i}{}^{*}\right)}{M_{\mathrm{cls}}}+w\frac{\sum_{i}p^{*}L_{\mathrm{reg}}\left(t_{i},t_{i}{}^{*}\right)}{M_{\mathrm{reg}}}$$

In Equation (3), i indicates an index of a mini-batch; $p_{i}$ is the probability of an anchor box or a proposal box including a face; $p_{i}{}^{*}$ is a ground-truth label, which if positive, becomes 1, and if negative, 0; $t_{i}$ is a bounding box regression vector of an anchor box or a proposal box; $t_{i}{}^{*}$ is a bounding box regression vector of a ground-truth; and $L_{\mathrm{cls}}$ is a classification loss function, and it indicates log loss of classes. The regression loss function ($L_{\mathrm{reg}}$) is a smooth L1 loss for regression, and this is only used when the anchor box or proposal box is positive ($p_{i}{}^{*}=1$). $M_{\mathrm{cls}}$ is the mini-batch size, and $M_{\mathrm{reg}}$ is the number of anchor boxes or proposal boxes. Two loss functions are normalized by using $M_{\mathrm{cls}}$ and $M_{\mathrm{reg}}$, and, finally, weights of the two loss functions are evenly adjusted through the balancing parameter, w.

### 5.3. Testing of Two-Step Faster R-CNN

#### 5.3.1. Comparative Experiments with RPN and Faster R-CNN in Body Detection Stage

In the first experiment, the method of using Faster R-CNN proposed in the body detection stage and the method using RPN without classifier were compared. Faster R-CNN can be divided into feature extractor, RPN, and classifier, and this experiment shows the difference in detection performance depending on the use of classifier. Accuracy is assessed by measuring the true positive rate (TPR) and precision as in Equations (4) and (5). TPR is also called recall. In Equations (4) and (5), #TP, #FP, and #FN indicate the numbers of true positives (TPs), false positives (FPs), and false negatives (FNs), respectively [57]. Here, positive and negative data represent the body (or face) and background, respectively; therefore, a false positive indicates an error case in which background is recognized as a body (or face), and false negative indicates an error case in which a body (or face) is recognized as background.

(4) $$\mathrm{TPR}\;(\mathrm{Recall})=\frac{\#TP}{\#TP+\#FN}.$$

(5) $$\mathrm{Precision}=\frac{\#\mathrm{TP}}{\#\mathrm{TP}+\#\mathrm{FP}}.$$

Table 3 shows the average accuracy obtained by performing the two-fold cross-validation at TPR (recall) and precision at the equal error rate (EER) point. EER indicates an error that occurs at the point where recall and precision are equal. Recall usually has trade-off characteristics with precision. That is, large recall by threshold of our system causes small precision, whereas small recall causes large precision. Therefore, we show the EER of precision and recall. EER means the recall or precision value when these values are same. EER value is that in the case that ROC curve is intersected with EER line. For example, in Figure 12b, the ROC curve by fine-tuned HR [17] (dark blue line) is intersected with the EER line (light blue and straight line) at the position of recall (0.9566) and precision (0.9566). Therefore, the recall and precision at the EER case are 0.9566 (95.66%) and 0.9566 (95.66%), respectively, as shown in Table 6. Because both recall and precision are same on this EER line as shown in these figures, the recall and precisions in Table 3, Table 4, Table 5, Table 6 and Table 7 are identical.

In Table 3, the body detection performance using the proposed step-1 Faster R-CNN is higher than when RPN is used alone.

**Figure 9:**
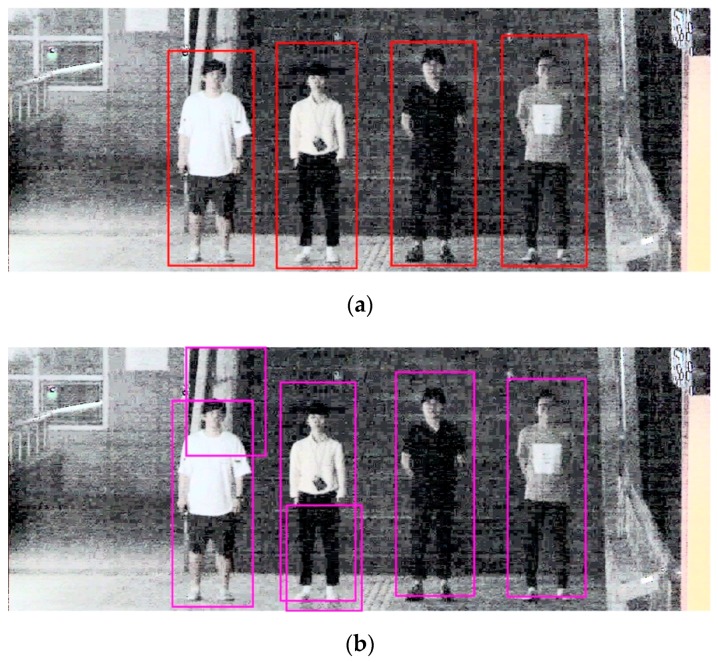
Examples of body detection results using: (**a**) Step-1 Faster R-CNN; and (**b**) RPN.

Figure 9 shows an example of body detection using step-1 Faster R-CNN and RPN. As shown in Figure 9b, when using RPN only, FPs that include a portion of the body are generated, degrading the detection performance. However, in step-1 Faster R-CNN, because the detection result of RPN is used as the region proposals and classification and regression are once again performed in the classifier, the FPs are reduced; thus, the detection performance is higher. In the detection process of the proposed method, because a low detection performance at the body detection stage affects the detection performance at the face detection stage, a high detection performance at the first stage is important. In this study, step-1 Faster R-CNN, which shows a higher performance than RPN only in the body detection stage, is used because we aim for a high face detection rate at night.

#### 5.3.2. Comparative Experiments with Original Nighttime Image and Histogram Equalization-Processed Image

In the second experiment, the face detection performance of the Two-Step Faster R-CNN using the original night image as an input was compared with the performance using the HE-processed image as an input. A two-fold cross-validation is performed for a fair performance evaluation. Table 4 presents the EER performance of each fold and the average recall and precision obtained from the two-fold cross-validation. As indicated in Table 4, the proposed method using the HE-processed images shows a higher face detection performance than using the original night images.

**Figure 10:**
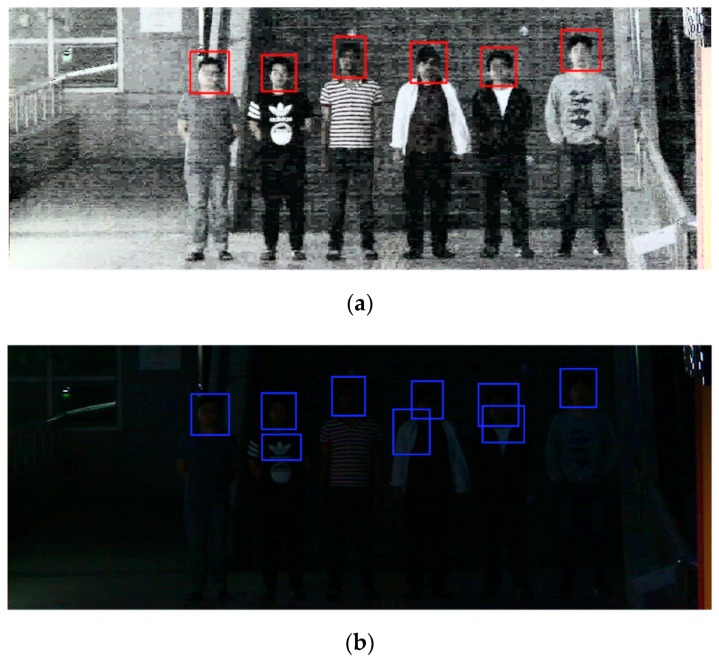
Example of face detection results using Two-Step Faster R-CNN: (**a**) test results using HE-processed image; and (**b**) test results using original nighttime image.

Figure 10 shows an example of face detection using the Two-Step Faster R-CNN. When the original night image is used, as shown in Figure 10b, FPs are produced around a complicated background or face. It is difficult to extract good features that can distinguish the face and the background because the intensity of illumination and contrast in the original night image are extremely low that they cannot even be distinguished by human eyes. However, because the contrast of the face and the background is increased through the normalization of HE in the HE-processed image, the detection accuracy could be enhanced, as shown in Figure 10a.

#### 5.3.3. Comparative Experiments with Two-Step Faster R-CNN and Single Faster R-CNN

In the third experiment, the method of night face detection using the Two-Step Faster R-CNN and the method of direct face detection without body detection in the input image using single Faster R-CNN are compared. Because the experiment described in Section 5.3.2 proved that the detection performance is improved by preprocessing an image with the HE method, both methods compared in this experiment use the input image preprocessed by the HE method. Furthermore, for a fair comparison, when the face is directly detected in the image using single Faster R-CNN, considering the size and ratio of faces in the image, nine different anchor boxes of three scales (64 × 64, 128 × 128, and 256 × 256) and three aspect ratios (1:1, 1:1.2, and 1:4) are used. Table 5 presents the EER performance of each fold and the average recall and precision obtained by the two-fold cross-validation using the proposed method and the single Faster R-CNN. In Table 5, the face detection performance of the Two-Step Faster R-CNN is higher than that of the single Faster R-CNN. Because the single Faster R-CNN detects faces directly in the entire input image, the number of FPs generated in the same TPR is much larger than that of Two-Step Faster R-CNN.

**Figure 11:**
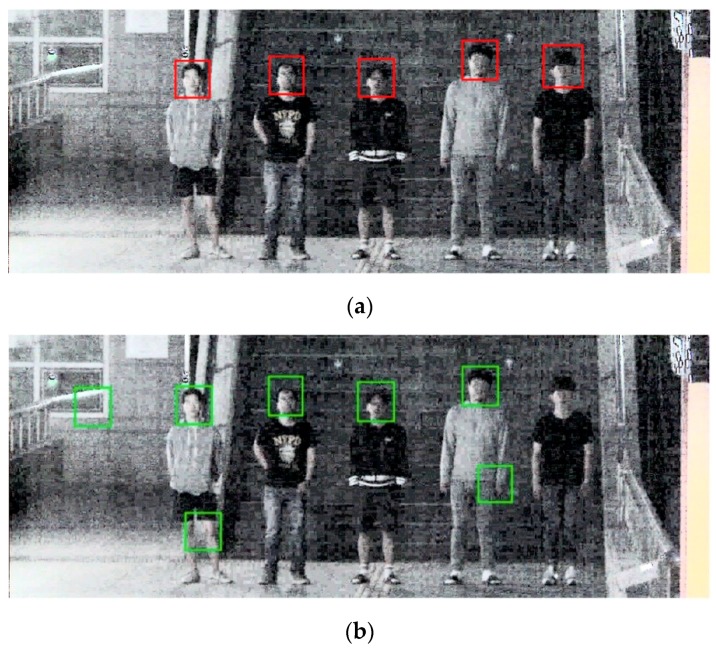
Example of face detection results using Two-Step Faster R-CNN and single Faster R-CNN: (**a**) test result using Two-Step Faster R-CNN; and (**b**) test results using single Faster R-CNN.

Figure 11 shows an example of face detection using the Two-Step Faster R-CNN and single Faster R-CNN. Figure 11b shows the test result using a single Faster R-CNN; it shows FPs containing complex backgrounds or a portion of a body generated, and FN is also generated. However, as shown in Figure 11a, in the case of the proposed method, Two-Step Faster R-CNN, the number of FPs is reduced by detecting the body first and then detecting faces only within the upper body region of the detected body. Therefore, the proposed method has a higher performance.

#### 5.3.4. Comparative Experiments of Proposed Method with Previous Methods

##### Descriptions of Previous Methods

In the next experiment, the nighttime face detection performance using the existing face detection methods in [1,16,17,18,19] and the proposed method were compared and analyzed. Adaboost [1] and the NPD face detector (NPDFace) [16] are based on hand-crafted features, whereas the hybrid resolution (HR)-based method [17], multitask cascaded convolutional networks (MTCNN) [18] and you only look once version 2 (YOLOv2) [19] are CNN-based methods. 

NPDFace [16] detects faces using the adaboost algorithm based on the NPD feature, which represents the ratio of the difference between two pixel values to the sum of those two pixels. MTCNN [18] is a cascaded CNN framework consisting of three stages: a proposal network (P-Net) that finds face candidates, a refine network (R-Net) that eliminates false candidates, and an output network (O-Net), which is similar to a refine network to some extent and which outputs the final detection results and facial landmarks’ positions. For HR [17] and YOLOv2 [19], the fine-tuning performance is measured. There are two kinds of training scheme. The first one is “training from scratch” and the second one is “fine-tuning (transfer learning)” [58]. In the former method, the whole network including parameters and weights are trained with domain-specific database (in our research, self-collected DNFD-DB1 and open database of Fudan University [49]). However, in the latter method, the whole network including parameters and weights are first trained with a different database (this is called as pretrained model), and only the parts of parameters and weights of the pretrained model are trained again with domain-specific database (in our research, self-collected DNFD-DB1 and open database of Fudan University [49]). For comparisons, the YOLOv2 [19] and HR [17] (pretrained with their databases, respectively) are fine-tuned with our experimental databases of DNFD-DB1 and open database of Fudan University, respectively. In addition, we compared the accuracies by our method with those by the simplified approach (body detection by our step-1 Faster R-CNN with YOLO-based face detection for the two-step detector). For convenience, we present this simplified approach as “Step-1 Faster R-CNN + Fine-tuned YOLOv2”. YOLOv2 [19] is a single convolutional network using darknet-19 as a feature extractor, which simultaneously predicts multiple bounding boxes and class probabilities in an input image. First, an input image is rescaled, and high level feature maps are generated through the feature extractor and concatenated with low level feature maps from an earlier layer. Finally, through the last convolutional layer, multiple bounding boxes and class probabilities are produced. In [19], YOLOv2 showed higher performance and faster detection speed than the other detection methods through various detection datasets. Hybrid resolution (HR) [17] is a resnet101-based FCN model for detecting both large-size faces and very small-size faces. The input is an image pyramid created using rescaled images, and scale-variant templates are applied to multiscale features extracted from multiple layers (last layer of res-blocks) of FCN-resnet101 to obtain response maps. Finally, NMS is applied to the result detected in each image pyramid to produce the final detection result. HR [17] showed a high face detection performance using multiscale features containing large receptive fields and additional context information. 

The image preprocessed by histogram equalization for CNN input is also the part newly proposed in our method and the existing algorithms do not use preprocessed image (see [1,16,17,18,19]). Therefore, the original image without preprocessing was used for other algorithms [1,16,17,18,19]. The source codes of other algorithms with parameters were obtained from the websites provided by the authors of [1,16,17,18,19].

##### Comparative Experiments

**Figure 12:**
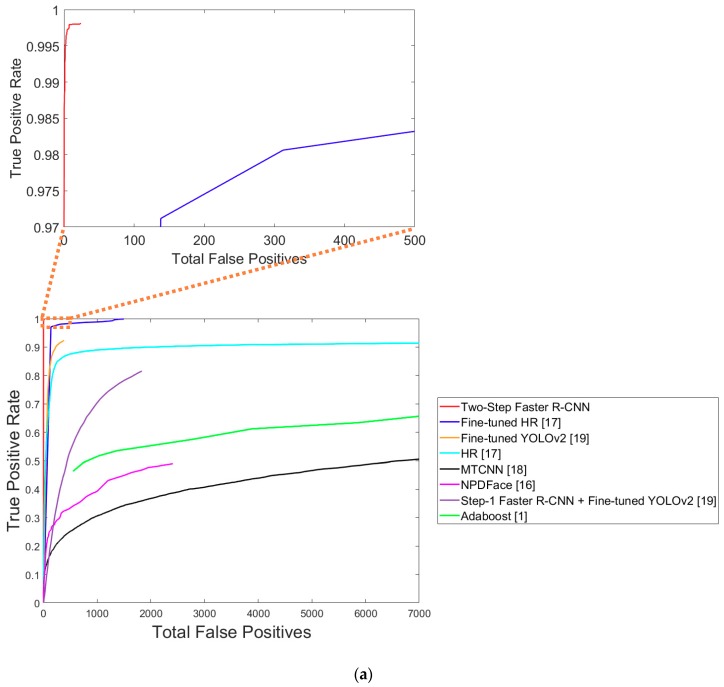

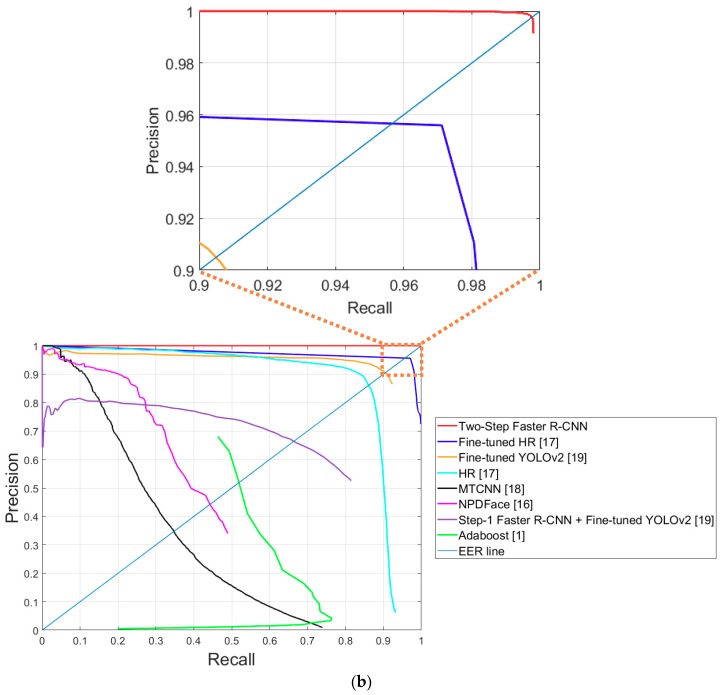
Nighttime face detection performance of existing methods and the proposed method using DNFD-DB1: (**a**) True positive rate (TPR) curves according to total FPs; and (**b**) receiver operating characteristic (ROC) curve of recall and precision.

Table 6 presents the average recall and precision EER performance of the proposed method using DNFD-DB1 and the existing methods. Figure 12 shows both the TPR according to the total number of FPs and the receiver operating characteristic (ROC) curves between recall and precision, obtained by applying the detection score of the face detectors used in the comparative experiment as a threshold. For stochastic analysis, we randomly selected data from testing Subset 1 and Subset 2 of Table 2 five times, respectively, and obtained the average accuracy and standard deviation of accuracy from 10 trials as shown in Table 6. Figure 12 is the average graph of 10 trials. Table 6 and Figure 12 show that the proposed method produces significantly fewer FPs than the other methods and it has the highest detection performance.

**Figure 13:**
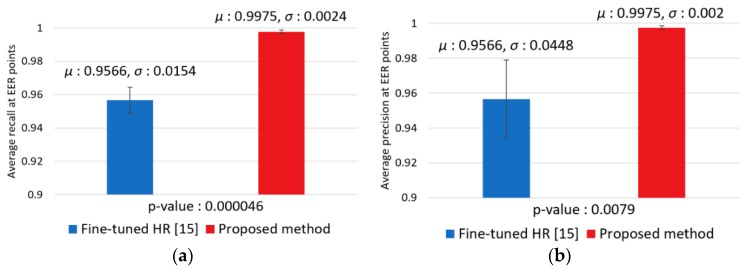
*T*-test with the accuracies (EER of: (**a**) recall; and (**b**) precision) by our method and the second best method (fine-tuned HR).

We performed a *t*-test [59] for showing the significance of our method as shown in Figure 13. In null hypothesis for t-test, it is assumed that there is no difference between the accuracy of our method and that of the second best method (fine-tuned HR [17] of Table 6 and Figure 12). Experimental results in Figure 13 show that the *p*-values of recall and precision for this t-test was 0.000046 and 0.0079 (less than 0.01), respectively, which show that the null hypothesis is rejected at a 99% confidence level indicating that there is a significant difference at a 99% confidence level between the accuracies (recall and precision) by our method and those by the second best method.

In addition, for analyzing the reliability of the observed phenomena in descriptive statistics, we used the Cohen’s *d* method [60,61]. It is calculated based on the average difference between the accuracy of our method and that of the second best method which is divided by standard deviation. Strength or effect sizes such as small, medium, and large are defined by Cohen’s *d* values of 0.2, 0.5, and 0.8 respectively. Experimental results in Figure 13 show the Cohen’s *d* values of 3.7 (recall) and 1.29 (precision), respectively. Because these Cohen’s *d* values are close to 0.8, the results present the differences between the accuracy of our method and that of the second best method are large in effect size.

**Figure 14:**
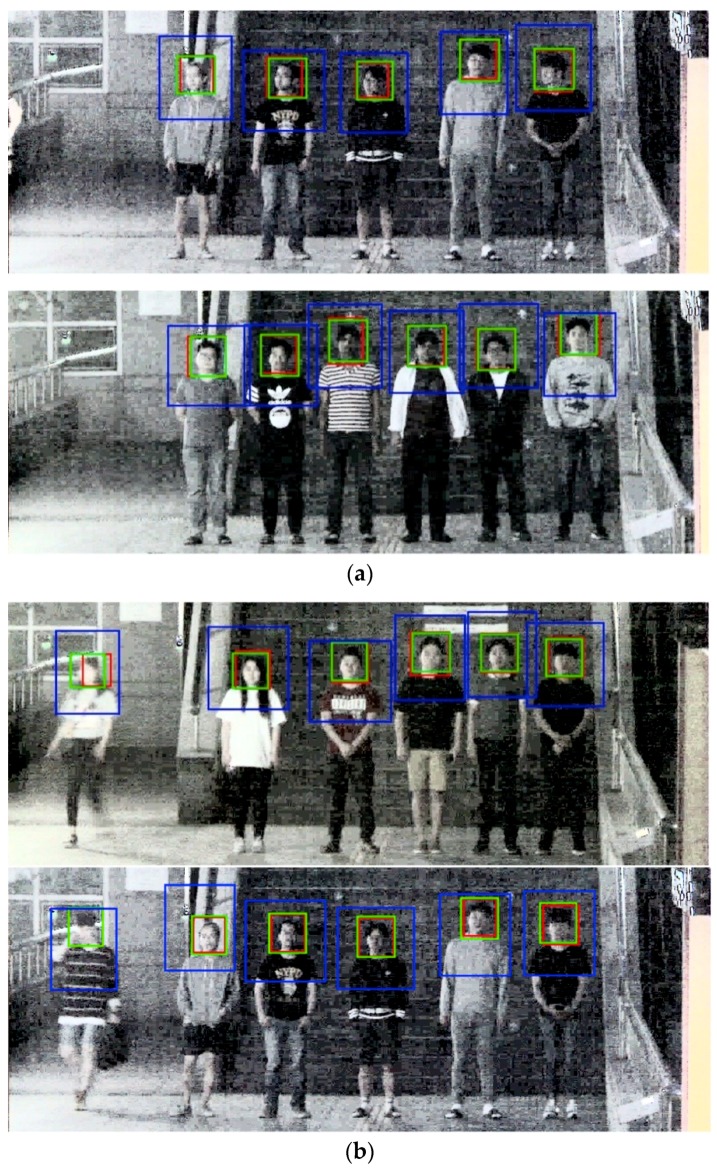
Example image of DNFD-DB1 night face detection using Two-Step Faster R-CNN: (**a**) correct detection cases; and (**b**) error cases. (The red box is the detection box, the blue box is the upper body detection box, and the green box is the ground-truth.)

Figure 14a shows DNFD-DB1 test result images with good nighttime face detections. In addition to the face from frontal view, the side view is also detected very well. Moreover, the face is well detected even when noise and blur levels are high. Figure 14b shows nighttime face detection error images, and FN error tends to be generated when motion blur is severe.

#### 5.3.5. Comparative Experiments of Proposed Method with Previous Methods Using Open Database

**Figure 15:**
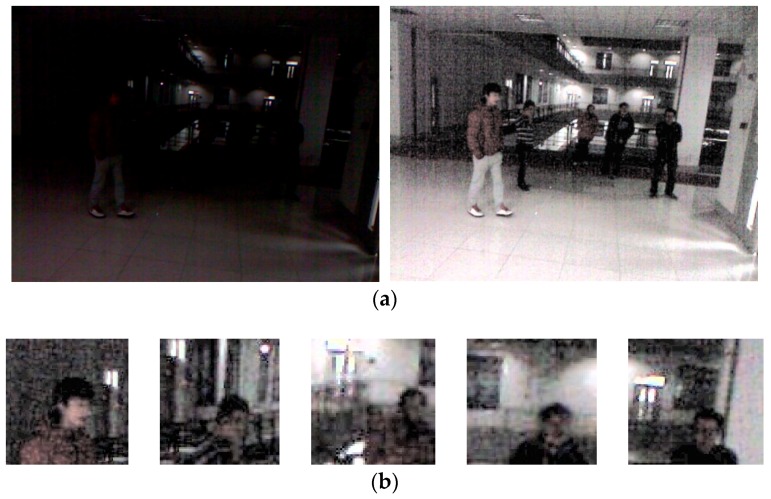
Examples of images in open database used for experiments: (**a**) images of the open database (the image on the left is the original image, and the image on the right is the HE-processed image); and (**b**) upper body images of the open database.

As an additional experiment, the proposed method and existing face detection methods were compared using the open database of Fudan University [49]. The open database contains images captured at Fudan University in a low light environment. The resolution of the camera is 640 × 480. The images were captured of sic different people with 4–6 people per frame. The Two-Step Faster R-CNN learned with DNFD-DB1 is fine-tuned using training set images in the open database. Figure 15a shows the original image and the HE-processed image of the open database, and Figure 15b shows the images of the upper body area cropped from the body detected by the step-1 Faster R-CNN. In Figure 15a, the right image is used for fine-tuning in step-1 Faster R-CNN, and the images in Figure 15b are used for fine-tuning in Two-Step Faster R-CNN.

**Figure 16:**
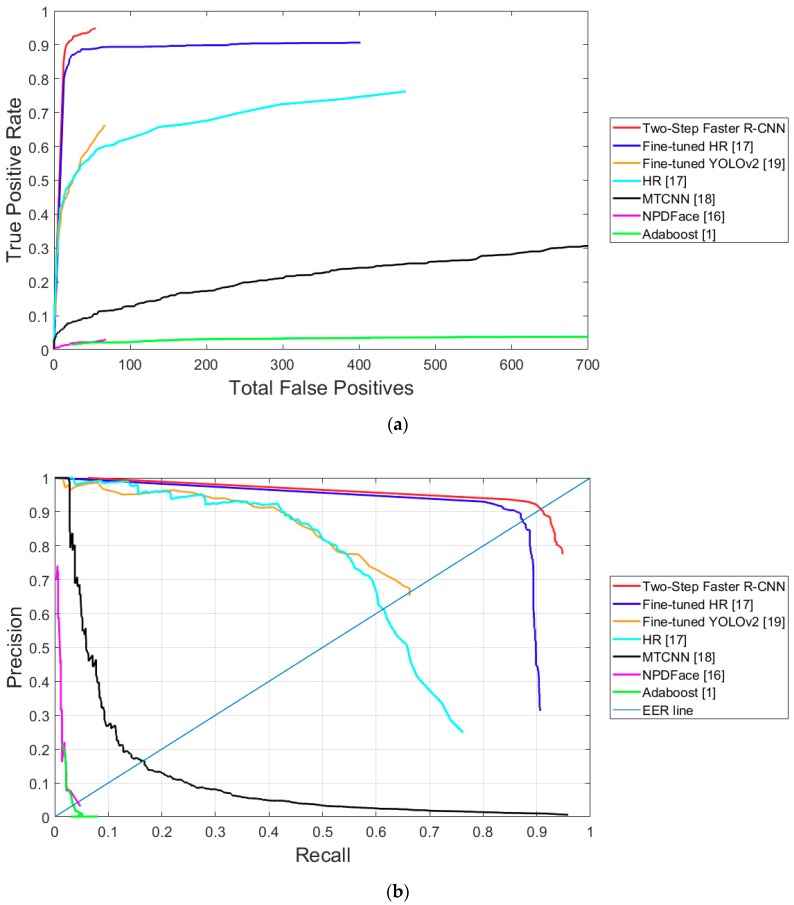
Graphs of nighttime face detection performances of existing methods and proposed method using the open database: (**a**) TPR curves according to total FPs; and (**b**) ROC curve of recall and precision.

Table 7 presents the average recall and precision performances of the proposed method and the existing methods using the open database. Similar to the results in Table 6, for stochastic analysis, we randomly selected data from testing Subset 1 and Subset 2 in Table 2 five times, respectively, and obtained the average accuracy and standard deviation of accuracy from 10 trials, as shown in Table 7. Figure 16 is the average graph of 10 trials. Figure 16 shows both the TPR according to the total number of FPs and the ROC curves between recall and precision, obtained by applying the detection score of the face detectors used in the comparative experiment as a threshold. Table 7 and Figure 16 show that the proposed method has a better performance than the other methods.

**Figure 17:**
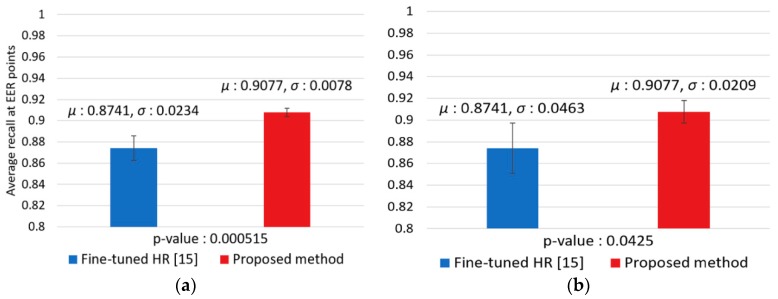
T-test with the accuracies (EER of: (**a**) recall; and (**b**) precision) by our method and the second best method (fine-tuned HR).

We performed a t-test to show the significance of our method (Figure 17). Experimental results in Figure 17 show that the *p*-value of recall and precision for this t-test was 0.000515 (less than 0.01) and 0.0425 (less than 0.05), respectively. These results show that the null hypothesis for recall is rejected at a 99% confidence level indicating that there is a significant difference between the recall of our method and that of the second best method. In addition, the null hypothesis for precision is rejected at a 95% confidence level indicating that there is a significant difference between the precision of our method and that of the second best method. In addition, for analyzing the reliability of the observed phenomena in descriptive statistics, we used the Cohen’s *d* method. Experimental results in Figure 17 show the Cohen’s *d* values of 1.92 (recall) and 0.93 (precision), respectively. Because all these Cohen’s *d* values are the closet to 0.8, these results represent the differences between the accuracy of our method and that of the second best method are large in effect size.

**Figure 18:**
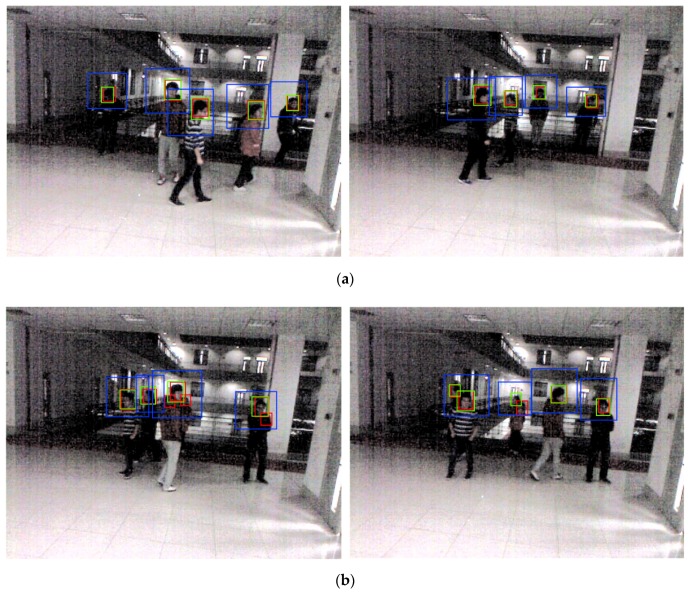
Example image of nighttime face detection by Two-Step Faster R-CNN using the open database: (**a**) correct detection cases; and (**b**) error cases. (The red box is the detection box, the blue box is the upper body detection box, and the green box is the ground-truth.)

Figure 18a shows the open database test results with good nighttime face detections; it also detects faces in side view as well as faces in frontal view in an environment with high noise and blur. Figure 18b shows nighttime face detection error images. When noise, blur, and occlusion levels are extremely high or face size is extremely small, detection errors occur.

#### 5.3.6. Analyses

In the comparative experiments using DNFD-DB1 and the open database, we confirmed that the proposed Two-Step Faster R-CNN has a better performance than the existing face detection methods [1,16,17,18,19]. The existing methods [1,16,17,18,19] had a lower detection performance than the Two-Step Faster R-CNN because databases in different environments were used. Because DNFD-DB1 and open database are images of visible-light taken at night, there is a lot of noise and blur. In such a nighttime environment, it is difficult to extract features that can distinguish the face from the background, because the contrast between the face and background is low and the boundary is ambiguous. In the comparative experiment of this study, the test results of fine-tuned YOLOv2 [19] also showed high recall and precision of 90.49%, but it is lower than the proposed method showing the recall and precision of 99.75%. Because the distinction between face and background is not clear in the night image, the bounding box regression of YOLOv2 cannot obtain the correct face area. HR [17] is difficult to detect the accurate face area by using large context information because the nighttime databases using in this study are low in contrast and the distinction between the face and background is not clear owing to high noise and blur. The test results of fine-tuned HR [17] with DNFD-DB1 and open database showed a better performance than HR without being fine-tuned, but the performance is still lower than that of the proposed Two-Step Faster R-CNN. In the case of adaboost [1], the reason for the poor performance using the nighttime database is that Haar-like features are used. Because Haar-like features are defined as the difference of the sum of pixels within a rectangular region, the nighttime images with small pixel brightness values within the region and low contrast have small feature values. Furthermore, it is difficult to select Haar-like features that can express facial features due to noise and blur. Because the number of layers in MTCNN [18] is smaller than that of Faster R-CNN networks, it would be difficult to extract features of faces in nighttime images sufficiently. In addition, because the input of MTCNN [18] uses an image pyramid and P-Net generates candidates with 12 × 12 windows with a single ratio, it is possible that the performance is degraded due to low illumination, high noise and blur in a nighttime database. In a nighttime database, face detection by NPDFace [16] is difficult because the boundary between the face and background is ambiguous and the brightness difference between pixels is small. Compared to the other methods [1,16,17,18,19], Two-Step Faster R-CNN increases the detection accuracy by detecting the body region first and then detecting the face in the upper body region. As a result, the proposed method showed a higher detection performance compared to the existing methods when applied to the DNFD-DB1 and the open database.

As the last experiment, we compared the computational performances (average processing time per each image) by our method and previous methods. Experiments were performed on the desktop computer explained at the end of Section 5.1. As shown in Table 8, although the processing time by our method is longer than MTCNN [18], NPDFace [16], Adaboost [1], Fine-tuned YOLOv2 [19], and step-1 Faster R-CNN + Fine-tuned YOLOv2 [19], our accuracies are higher than these methods, as shown in Table 6 and Table 7 and Figure 12, Figure 13, Figure 16 and Figure 17. In addition, we implemented our algorithm by Matlab Caffe (version 1) [51] with Matlab (version 2017a) [52] as explained at the end of Section 5.1. Therefore, if we were to implement our algorithm in Darknet [62], as YOLOv2 [19], or Linux (or Windows) Caffe [63] based on C programming in the future, we can reduce the processing time of our method greatly with the same accuracy.

## 6. Conclusions

In this study, a face detection method using visible-light images taken at nighttime was investigated. It is difficult to detect the face in a visible-light image taken at nighttime because it has a low intensity of illumination. By applying HE in the preprocessing, the visibility of the face was improved owing to the increased contrast between the face and the background, and the enhanced detection performance was proved through experiments. In the detection step, the body was detected first in the input image using the Two-Step Faster R-CNN model, and the face was then detected in the upper body region. It was shown that that detection performance can be enhanced by using this two-step detection method and compared to other face detectors, the proposed method showed a higher performance in the comparative experiments in this study. In addition, a self-constructed database (DNFD-DB1) and a learned Two-Step Faster R-CNN model made from images acquired with a single visible-light camera at night were presented in [36] so that other researchers can access them. Experimental results also showed that most face detection errors are caused by too much noise, blur, or occlusion of a part of the face.

In the future, a method of improving face detection performance in combination with optical and motion blurring restoration, super-resolution reconstruction, and denoising methods for far and night input images will be investigated. Furthermore, a method of improving the detection accuracy by compensating occluded faces based on generative adversarial network will be researched. To shorten the training time and reduce the complexity of the computation, a method of maintaining the detection accuracy with a reduced number of layers and filters in the proposed Two-Step Faster R-CNN will be studied as well.

## Figures and Tables

**Table 1:** Comparison of previous studies and proposed method on face detection.

Category		Method	Advantages	Disadvantages
Multiple camera-based method		Dual-band system of NIR and SWIR cameras [6]	- NIR and SWIR cameras are robust to illumination changes and low light intensity. - The algorithm is not complicated because of image fusion method.	- A calibration between cameras is necessary. - The intensity and angle of IR illuminator need to be adjusted according to its distance from the object.
Single camera-based methods	Using thermal camera	Multi-slit method [7], Haar + LBP [8], Haar + HOG + AMB-LTP [9]	- A thermal camera is robust to illumination changes and low light intensity. - Complicated computation is not required [7]. - Facial features in thermal images are used [9].	- A thermal camera is expensive. - It is difficult to detect faces in an environment where the background and human temperatures are similar. - If the position and angle of camera change, the parameters need to be updated [7].
	Using NIR or SWIR camera	Three adaboost cascades [10], occupant detection with adaboost [11], cascade pattern detector [12], adaboost + FCN [13], Viola-Jones face detector [22], manually detected (or detected by commercial software) face region [32,33,34,35]	- NIR and SWIR cameras are robust to illumination changes and low light intensity. - Three adaboost cascades are used to consider changes in the driver’s facial pose [10].	The intensity and angle of IR illuminator need to be adjusted according to its distance from the object.
	Using visible-light camera	Hybrid skin segmentation [23], region-based skin-color segmentation [24], adaboost with PRO-NPD features [25], face detection using GA [26], RASW-based Viola-Jones face detector [31], manually detected (or detected by commercial software) face region [32,33,34,35]	The price of camera is low.	- Performance is low at night when little color information is available and the noise level is high [23,24,25,31]. - Multiple faces cannot be detected [26].
		Image enhancement for face detection [29,30]	- The contrast of night image is enhanced to increase the visibility of faces.	- Noise level increases with increased visibility. - Processing time increases due to preprocessing.
		Two-Step Faster R-CNN (**proposed method**)	- Accuracy is improved through a two-step detection. - Deep learning-based features improve detection performance even with high noise or blur.	Training data and time to learn CNN are required.

NIR: near-infrared; SWIR: short-wave IR; LBP: local binary pattern; HOG: histogram of oriented gradients; IR: infrared; AMB-LTP: absolute multiblock local ternary pattern; FCN: fully convolutional network; PRO-NPD: promotion normalized pixel difference; GA: genetic algorithm; RASW: run-time adaptive sliding window; R-CNN: region-based convolutional neural network.

**Table 2:** Description of Dongguk Nighttime Face Detection database (DNFD-DB1).

DNFD-DB1	Subset 1	Subset 2
Number of people	10	10
Number of images	848	1154
Number of augmented images	1696	2308
Number of face annotations	4286	5809
Resolution (width × height) (pixels)	1600 × 600	
Width of face (min – max) (pixels)	45 − 80	
Height of face (min – max) (pixels)	48 − 86	
Environment of database	- Images were obtained using a visible-light camera in a surveillance camera environment. - The height of the camera is approximately 2.3 m from the ground, and the distance from a person is approximately 20–22 m. - Images were taken at night environment of approximately 10–20 lux (at 9–10 pm).	

**Table 3:** Two-fold cross-validation results for body detection at equal error rate (EER) of recall and precision (unit: %).

Models	DNFD-DB1 Subsets	Recall	Precision	Average Recall	Average Precision
RPN	1st fold	99.16	99.16	98.34	98.34
	2nd fold	97.52	97.52		
Step-1 Faster R-CNN	1st fold	99.97	99.97	99.94	99.94
	2nd fold	99.91	99.91		

**Table 4:** Two-fold cross-validation results with and without preprocessing at EER points of recall and precision (unit: %).

Input Image	DNFD-DB1 Subsets	Recall	Precision	Average Recall	Average Precision
Original nighttime image (without preprocessing)	1st fold	98.83	98.83	98.50	98.50
	2nd fold	98.17	98.17		
HE-processed image	1st fold	99.89	99.89	99.76	99.76
	2nd fold	99.63	99.63		

**Table 5:** Two-fold cross-validation results of Two-Step Faster R-CNN and single Faster R-CNN at EER points of recall and precision (unit: %) (#FP and #FN are the average numbers of false positive and false negative from two-fold cross validation, respectively).

Methods	DNFD-DB1 Subsets	Recall	Precision	Average Recall	Average Precision	#FP	#FN
Single Faster R-CNN	1st fold	79.93	79.93	79.04	79.04	2115.9	2115.9
	2nd fold	78.15	78.15				
Two-Step Faster R-CNN	1st fold	99.89	99.89	99.76	99.76	24.2	24.2
	2nd fold	99.63	99.63				

**Table 6:** Performance comparison between existing and proposed methods at EER points of recall and precision (unit: %). (avg. and std. mean average value and standard deviation value, respectively) (#FP and #FN are the average numbers of false positive and false negative from 10 trials, respectively).

Methods	Recall (avg.(std.))	Precision (avg.(std.))	#FP	#FN
MTCNN [18]	34.74 (0.0834)	34.74 (0.0211)	1579.4	1579.4
NPDFace [16]	44.26 (0.0126)	44.26 (0.0506)	1345.4	1345.4
Adaboost [1]	51.88 (0.0143)	51.88 (0.0225)	1029.8	1029.8
Step-1 Faster R-CNN + Fine-tuned YOLOv2 [19]	66.36 (0.0363)	66.36 (0.0182)	862.5	862.5
HR [17]	86.12 (0.0216)	86.12 (0.0360)	338.8	338.8
Fine-tuned YOLOv2 [19]	90.49 (0.0087)	90.49 (0.0166)	251.2	251.2
Fine-tuned HR [17]	95.66 (0.0154)	95.66 (0.0448)	137.9	137.9
Two-Step Faster R-CNN (proposed method)	99.75 (0.0024)	99.75 (0.0020)	6.9	6.9

**Table 7:** Average performance of existing methods and proposed methods using the open database at EER points of recall and precision (unit: %) (avg. and std. mean average value and standard deviation value, respectively).

Methods	Recall (avg.(std.))	Precision (avg.(std.))
Adaboost [1]	3.43 (0.0098)	3.43 (0.0102)
NPDFace [16]	4.18 (0.0177)	4.18 (0.0348)
MTCNN [18]	16.53 (0.0361)	16.53 (0.0277)
HR [17]	61.31 (0.0798)	61.31 (0.0430)
Fine-tuned YOLOv2 [19]	66.23 (0.0255)	66.23 (0.0462)
Fine-tuned HR [17]	87.41 (0.0234)	87.41 (0.0463)
Two-Step Faster R-CNN (Proposed method)	90.77 (0.0078)	90.77 (0.0209)

**Table 8:** Comparisons of the computational performances (average processing time per each image) by our method and previous methods (unit: ms).

Methods	Processing Time
MTCNN [18]	122
NPDFace [16]	47
Adaboost [1]	70
HR [17]	1182
Fine-tuned YOLOv2 [19]	23
Fine-tuned HR [17]	1182
Step-1 Faster R-CNN + Fine-tuned YOLOv2 [19]	98.4
Two-Step Faster R-CNN (proposed method)	315

## Appendix A

**Table A1:** Architecture of feature extractor of Figure 3.

Layer Type	Number of Filters	Size of Feature Map (Height × Width × Channel)	Size of Kernel (Height × Width × Channel)	Number of Strides	Number of Paddings
Input layer [image]		300 × 800 × 3			
Conv1_1 (1st convolutional layer)	64	300 × 800 × 64	3 × 3 × 3	1 × 1	1 × 1
Relu1_1		300 × 800 × 64			
Conv1_2 (2nd convolutional layer)	64	300 × 800 × 64	3 × 3 × 64	1 × 1	1 × 1
Relu1_2		300 × 800 × 64			
Max pooling layer	1	150 × 400 × 64	2 × 2 × 1	2 × 2	0 × 0
Conv2_1 (3rd convolutional layer)	128	150 × 400 × 128	3 × 3 × 64	1 × 1	1 × 1
Relu2_1		150 × 400 × 128			
Conv2_2 (4th convolutional layer)	128	150 × 400 × 128	3 × 3 × 128	1 × 1	1 × 1
Relu2_2		150 × 400 × 128			
Max pooling layer	1	75 × 200 × 128	2 × 2 × 1	2 × 2	0 × 0
Conv3_1 (5th convolutional layer)	256	75 × 200 × 256	3 × 3 × 128	1 × 1	1 × 1
Relu3_1		75 × 200 × 256			
Conv3_2 (6th convolutional layer)	256	75 × 200 × 256	3 × 3 × 256	1 × 1	1 × 1
Relu3_2		75 × 200 × 256			
Conv3_3 (7th convolutional layer)	256	75 × 200 × 256	3 × 3 × 256	1 × 1	1 × 1
Relu3_3		75 × 200 × 256			
Max pooling layer	1	38 × 100 × 256	2 × 2 × 1	2 × 2	0 × 0
Conv4_1 (8th convolutional layer)	512	38 × 100 × 512	3 × 3 × 256	1 × 1	1 × 1
Relu4_1		38 × 100 × 512			
Conv4_2 (9th convolutional layer)	512	38 × 100 × 512	3 × 3 × 512	1 × 1	1 × 1
Relu4_2		38 × 100 × 512			
Conv4_3 (10th convolutional layer)	512	38 × 100 × 512	3 × 3 × 512	1 × 1	1 × 1
Relu4_3		38 × 100 × 512			
Max pooling layer	1	19 × 50 × 512	2 × 2 × 1	2 × 2	0 × 0
Conv5_1 (11th convolutional layer)	512	19 × 50 × 512	3 × 3 × 512	1 × 1	1 × 1
Relu5_1		19 × 50 × 512			
Conv5_2 (12th convolutional layer)	512	19 × 50 × 512	3 × 3 × 512	1 × 1	1 × 1
Relu5_2		19 × 50 × 512			
Conv5_3 (13th convolutional layer)	512	19 × 50 × 512	3 × 3 × 512	1 × 1	1 × 1
Relu5_3		19 × 50 × 512			

**Table A2:** Architecture of region proposal network (RPN) of Figure 3.

Layer Type	Number of Filters	Size of Feature Map (Height × Width × Channel)	Size of Kernel (Height × Width × Channel)	Number of Strides	Number of Paddings
Input layer [Conv5_3]		19 × 50 × 512			
Conv6 (14th convolutional layer) Relu6	512	19 × 50 × 512 19 × 50 × 512	3 × 3 × 512	1 × 1	1 × 1
Classification (convolutional layer) Softmax	18	19 × 50 × 18 19 × 50 × 18	1 × 1 × 512	1 × 1	0 × 0
Regression (convolutional layer)	36	19 × 50 × 36	1 × 1 × 512	1 × 1	0 × 0

**Table A3:** Architecture of classifier of Figure 3. (From the ROI pooling layer, the processed results of the proposals are displayed instead of the entire input image; * denotes the coordinates of the proposals (*x*_min, *y*_min, *x*_max, and *y*_max); ** denotes the probability of each face and background.)

Layer Type	Size of Output
Input layer	
[Conv5_3]	19 × 50 × 512
[region proposals]	300 × 4 ^*^
ROI pooling layer	7 × 7 × 512 × 300
Fc6 (1st fully connected layer)	4096 × 300
Relu6	4096 × 300
Dropout6	4096 × 300
Fc7 (2nd fully connected layer)	4096 × 300
Relu7	4096 × 300
Dropout7	4096 × 300
Classification (fully connected layer)	2 ^**^ × 300
Softmax	2 × 300
Regression (fully connected layer)	4 × 300
